# An In Vitro Comparison of Coronal Microleakage of Three Orifice Barriers Filling Materials

**Published:** 2012-08-01

**Authors:** Hamidreza Yavari, Mohammad Samiei, Mahsa Eskandarinezhad, Shahriar Shahi, Marzieh Aghazadeh, Yones Pasvey

**Affiliations:** 1. Department of Endodontics, Dental School, Tabriz University of Medical Sciences, Tabriz, Iran; 2. Dental and Periodontal Research Center, Dental School, Tabriz University of Medical Sciences, Tabriz, Iran; 3. Department of Oral Medicine, Dental School, Tabriz University of Medical Sciences, Tabriz, Iran; 4. Private Practice, Tabriz, Iran

**Keywords:** Composite Resin, Glass-Ionomer, Microleakage, MTA, Root Canal Therapy

## Abstract

**Introduction:**

A coronal barrier in root-filled teeth is one of the most effective methods for prevention of coronal microleakage. The aim of this study was to compare coronal microleakage of three materials [light-cured glass-ionomer (GI), mineral trioxide aggregate (MTA), and composite resin] as coronal barriers.

**Materials and Methods:**

A total of 188 intact maxillary incisors were used. After instrumentation, all the canals were obturated with gutta-percha and lateral condensation technique using AH26 sealer. Then, the teeth were sectioned just apical to the cemento-enamel junction. The roots were randomly assigned to three experimental groups (n=56) and two negative and positive control groups (n=20). After placing the orifice barrier, the samples were immersed in 2% methylene blue solution for 2 weeks at 37°C. Then the teeth were longitudinally sectioned mesiodistally and dye penetration was measured under a stereomicroscope at ×10 magnification. Data were analyzed with one-way ANOVA and a post-hoc Tukey test.

**Results:**

The positive control group leaked significantly more than all the experimental groups (P=0.001). MTA exhibited less leakage than composite and GI (P=0.002) but no significant differences were found between GI and composite groups.

**Conclusion:**

Immediate placement of a suitable intra-orifice barrier like MTA, before final restoration, may help minimize recontamination of the remaining apical gutta-percha.

## Introduction

Bacteria and their products are the main cause of periapical inflammation [1][2]. Therefore, the chief aim of root canal treatment is to eliminate microorganisms from the root canal system and prevent re-infection [3][4]. Ray and Trope [5] reported that the quality of coronal restoration might be a more important factor than the quality of obturation in maintaining the periradicular health of the tooth. A hermetic seal after root canal treatment is needed to prevent bacteria from invading the peri-apex [6]. Lack of coronal seal might be the major cause of non-surgical endodontic failure [7]. The intra-orifice barrier is an efficient alternative method to decrease coronal leakage in endodontically treated teeth. This procedure includes placing additional material into the canal orifice immediately after removal of the coronal portion of gutta-percha and sealer [8]. Several materials have been used in an attempt to provide an intra-coronal seal to prevent microleakage, such as Cavit, amalgam, intermediate restorative material (IRM), Super-EBA, composite resin, glass-ionomer cement (GI), and mineral trioxide aggregate (MTA) [9][10].

Many materials such as Cavit, composite, IRM, and Super EBA [11][12][13] have been studied to determine their ability to seal endodontic access preparation [14][15]. Lee et al. reported that seal provided by Cavit is not durable against mastication forces; therefore, the search for a new temporary filling material has continued [16].

Zmener et al. showed no statistically significant differences in coronal leakage between Cavit, IRM, and Ultra Temp Firm as orifice barriers [17]. A further study showed that placement of 2 mm plugs of either bonded composite or IRM over gutta-percha obturations significantly reduced periapical inflammation [18].

Only glass-ionomer cement in the orifice may prevent bacterial penetration into the periapex compared to Cavit [6]. The sealing abilities of temporary filling materials as examined in many different studies have shown controversial results [6].

Recently, MTA (Dentsply Tulsa Dental, Tulsa, Ok, USA) was introduced as a root-end filling material in Endodontics. One reason that MTA has gained attention is its superior ability to resist leakage [19]. Such behavior might be explained by superior marginal adaptation of MTA [20]. Therefore, the aim of this study was to evaluate and compare microleakage between white MTA, light-cured GI, and composite resin as intra-orifice barriers using a dye penetration test.

## Materials and methods

In this experimental study, 188 extracted human central incisors were used. An access opening was prepared using a high-speed handpiece and a #2 round bur with constant water spray. After the pulp tissue was removed, working length was determined by measuring the length of a #10 file just visible at the apical foramen and subtracting 0.5 mm. The canals were instrumented to a #40 master file and tapered with a step-back technique.

Instrumentation was performed in conjunction with 2.5% NaOCl irrigation. Canals were dried with paper points. After instrumentation, all the canals were obturated with gutta-percha (Aria Dent, Tehran, Iran) and lateral condensation using AH26 sealer (DeTrey, Konstanz, Germany). Then, the teeth were sectioned just apical to the cemento-enamel junction with a low-speed diamond saw.

The roots were randomly assigned to three experimental groups with 56 samples each; 20 roots served as control (10 teeth as positive controls and 10 teeth as negative controls). The coronal aspect of the gutta-percha was adjusted to terminate 3 mm apical to the level of decoronation as measured by periodontal probe. The coronal 3 mm of the canal was cleaned of gutta-percha and sealer with an alcohol-moistened pellet, rinsed with sterile saline, and dried with an air stream.

The first group (56 teeth) received a 3 mm barrier of composite resin (Flow-It Alc Pentron Clinical, Dentsply, USA). The second group received light-cured GI (GC-Gold Label, Japan). The third group was sealed with white ProRoot MTA (Dentsply Tulsa Dental, Tulsa, Okla). All the materials were mixed and handled according to manufacturer’s instructions.

After placement of the test materials into access preparations, the specimens were stored in 100% relative humidity at 37°C for 48 hours. The specimens were thermocycled for 100 cycles in distilled water at 5°C/55°C, with a dwell time of 4 hours in each bath. All the specimens were submerged in molten sticky wax up to the CEJ to ensure an apical seal. Subsequently, the samples in the experimental groups and positive control group were coated with two layers of nail varnish except for 1 mm around the tooth-restoration interface. The positive control group consisted of 10 teeth obturated in the same manner as the experimental teeth without a coronal barrier. The negative control group consisted of 10 matching obturated teeth without coronal barrier, but with crowns and roots covered completely with nail varnish and sticky wax.

All the specimens were placed in 2% methylene blue solution with a pH value of 7.0 and stored for 2 weeks at 37°C. Then they were removed from dye solution and rinsed under tap water. After removal of the wax and nail varnish, the teeth were longitudinally sectioned in a mesiodistal direction with a low-speed diamond saw. The dye penetration was viewed using a stereomicroscope (Zeiss, Munich, Germany) at ×10 magnification and the degree of dye penetration was evaluated.

Data were analyzed by Kolmogorov-Smirnov test to indicate normal or non-normal distribution. As data had normal distribution one-way ANOVA was utilized for comparison followed by a post-hoc Tukey test. Statistical significance was defined at P<0.05.

## Results

All the materials leaked at the interface of restorative material-dentin wall. The mean leakage values (mm) and standard deviations (SD) are listed in Table 1.

**Table 1:** Results of microleakage of the three experimental and positive control groups (in mm)

**Groups**	**Mean (SD) **	**Number of teeth**
**MTA**	4.09 (0.85)	56
**Composite resin**	5.00 (1.36)	56
**Glass-ionomer**	5.02(1.15)	56
**Positive control**	12.43(3.23)	10

MTA had the lowest mean leakage value and the positive control group demonstrated the highest leakage. The negative control group showed no dye penetration. There were statistically significant differences between the positive control group and all the experimental groups (P<0.05); therefore, placement of a suitable intra-orifice barrier before final restoration is necessary to minimize the potential coronal microleakage. The Kolmogorov-Smirnov test revealed normal data distribution in MTA, composite resin, and GI samples. Therefore, one-way ANOVA was used for leakage comparison between the groups. The test results indicated significant differences between the four groups (P=0.000). The Tukey test revealed that this difference was only significant when comparing MTA with other groups (P=0.002), and there were no significant differences in pair-wise comparison of composite resin and GI (P=1.00).

## Discussion

The maintenance of a durable seal of the root canal system is necessary to minimize contamination of the root canal system during and after endodontic therapy [7][14][16][21].

Conventional root filling materials such as gutta-percha and sealer provide minimal resistance to bacterial microleakage [21][22]. Therefore, the coronal part of the root canal must be sealed as tightly as possible to minimize the endodontic treatment failure rate. Although previous research supports the effectiveness of intra-orifice barriers, there is no consensus as to the protocol or material used as the coronal barrier after root canal treatment [23][24]. Different authors have reported conflicting results about the sealing ability of different materials when used as a barrier [22][25][26]. Therefore, attempts are underway to introduce more qualified materials with the potential to provide a long-term seal.

Recently, MTA has been introduced for its superior ability to resist leakage when used as a barrier to augment the coronal seal or as a temporary restoration [19][26]. On the other hand, bonded resins and resin-modified glass ionomers (RMGI) seem to be promising materials to prevent coronal microleakage [25].

In the present study, GI, white MTA, and composite resin were compared. According to this study, all the experimental groups exhibited leakage within the materials. MTA showed the least coronal leakage, whereas GI showed the greatest coronal leakage compared to composite resin and MTA.

The results of this study are consistent with the results of another study [27], who reported that glass-ionomer leaked significantly more than MTA.

Cummings et al. compared MTA with IRM and ZnPO_4_ as a coronal barrier for internal bleaching [28]. Their results demonstrated that MTA had superior performance as a barrier.

In contrast, a study [29] reported no significant differences in leakage between Fuji Triage glass-ionomer and gray or white MTA. Yavari et al. showed that CEM cement and MTA, as intra-orifice sealing bio-materials, are more effective than amalgam and composite resin in preventing saliva leakage in endodontically treated teeth [30]. These favorable sealing properties, for the most part are related to their hydrophilic nature, good antibacterial potential, high pH, and formation of hydroxyapatite crystals in MTA and CEM cement materials [31][32][33]. Similar results were reported by Tselnik et al. [34]. They recommended Fuji П LC and MTA as an acceptable coronal seal up to 90 days. They believed that the superior performance of RMGI might be explained by water sorption by the material, resulting in setting expansion and consequently a better seal. RMGI requires no pre-treatment of dentin and can adhere to it. Another useful property of RMGI is the release of fluoride, which might decrease coronal microleakage through its antimicrobial activity [14][34][35][36].

All the studies differ in their design, making comparisons difficult. In a study by Tselnik, a bacterial-leakage model was used to evaluate coronal leakage, but in the present study, dye penetration was used. The dye penetration test is the most popular method of studying leakage, because it is easy to conduct, it is inexpensive, and it has a high degree of staining [37]. Molecules of dye have a low weight and can penetrate into locations that bacterial cells cannot [38]; therefore, in vitro microleakage studies with low-molecular-weight dyes or isotopes are more severe than those carried out in the mouth or with a clinically relevant macromolecular material [39]. Therefore, if cement resists dye penetration in vitro, it is likely to perform even better clinically. The limitation of dye leakage studies is that they measure the degree of leakage in only one plane, making it impossible to evaluate the total amount of leakage [40][41].

MTA might be suitable as an intra-orifice barrier because it has most of the ideal properties proposed by Wolcott et al., i.e. easy placement and excellent seal against microleakage [23].

Ease of removal of MTA for prosthetic concerns should be similar to colored glass-ionomer. MTA provide a good seal against microleakage [42] and has antimicrobial properties [43].

On the other hand, esthetic materials like composite resin, might potentially increase the possibility of perforation during restoration or re-entry into the canals due to their color matching ability [29].

In summary, immediate placement of a suitable intra-orifice barrier like MTA, before final restoration, could help minimize recontamination of the remaining apical gutta-percha.

## Conclusion

Within the limitation of this dye penetration technique study, MTA provided an acceptable coronal seal compared to GI and composite resin. Further in vivo studies on intra-orifice barriers are recommended.
